# Exploring Predictive Risk Factors of Infusion Reactions with First Pertuzumab Administration in HER2-positive Breast Cancer Patients: A Single Institution Experience

**DOI:** 10.31662/jmaj.2022-0132

**Published:** 2022-12-23

**Authors:** Kazutaka Otsuji, Masahiko Tanabe, Arisa Morizono, Mayumi Harada, Ayaka Sato, Takayoshi Niwa, Kotoe Nishioka, Yasuyuki Seto

**Affiliations:** 1 Department of Breast and Endocrine Surgery, Graduate School of Medicine, The University of Tokyo, Tokyo, Japan; 2 Next-Ganken Program, The Cancer Institute of Japanese Foundation for Cancer Research, Tokyo, Japan; 3 Department of Gastrointestinal Surgery, Graduate School of Medicine, The University of Tokyo, Tokyo, Japan

**Keywords:** breast cancer, HER2, pertuzumab, infusion reactions, risk factor

## Abstract

**Introduction:**

Pertuzumab and trastuzumab are monoclonal antibodies used for treating HER2-positive breast cancer. These anti-HER2 antibodies may induce infusion reactions (IR), mainly upon first administration. We investigated factors predicting IR in the initial pertuzumab treatment for HER2-positive breast cancer.

**Methods:**

We retrospectively reviewed the medical records of 57 patients who first received pertuzumab-containing treatment in our hospital from January 2014 to February 2021. The frequency of IR during or immediately after pertuzumab administration was examined. We also analyzed patient characteristics that may represent possible risk factors for IR.

**Results:**

The incidence rate of IR was 44% (25/57). Red blood cell count (P < 0.001), hemoglobin (Hb) concentration (P = 0.0011), and hematocrit (P < 0.001) immediately before pertuzumab administration were significantly lower in patients with IR than in those without. In patients with IR, erythrocyte levels immediately before pertuzumab treatment were significantly lower than baseline when having received anthracycline-containing chemotherapy within three months. Logistic regression analysis showed that a decrease in Hb levels was a significant risk factor for IR (log odds ratio = −17). According to the receiver-operating characteristic analysis, a 10% decrease in Hb after anthracycline-containing treatment was the best cut-off value for predicting IR (sensitivity: 88%; specificity: 77%; area under the curve: 0.87).

**Conclusions:**

Our study showed a higher incidence of IR after pertuzumab treatment than in clinical trials. There was a strong association between IR occurrence and erythrocyte levels lower than baseline in the group that received anthracycline-containing chemotherapy immediately before.

## Introduction

Human epidermal growth factor receptor 2 (HER2, which is encoded by the *ERBB2* gene), is amplified and/or over-expressed in 10%-25% of human breast cancers, being as it is a member of the epidermal growth factor receptor family. HER2 positivity in breast cancer is associated with aggressive metastatic disease and poor outcomes ^(1)^.

Pertuzumab (Pmab), an anti-HER2 monoclonal antibody, is used for treating HER2-positive metastatic breast cancer along with trastuzumab (Tmab), another anti-HER2 antibody ^(2), (3)^. Pmab has been available as neoadjuvant and adjuvant drug treatment for early HER2-positive breast cancer since 2017 ^(4)^; thus, its usage has been increasing.

These anti-HER2 antibodies may induce infusion reactions (IR), such as cytokine release syndrome, mainly upon first administration, where this IR is likely to result from an excessive immune response and do not necessarily require anti-drug antibodies. Although the exact mechanisms underlying IR are not fully elucidated, the IR symptoms may be biologically attributable to several factors, which include direct cytokine release, activation of the complement system and also activation of the coagulation system. IR symptoms typically manifest within 30-120 min after the injection has been initiated ^(5)^. In contrast, anaphylactic reactions usually occur very rapidly, within seconds or minutes after the start of infusion.

Clinical trial data for Pmab describe IR in more than 13% of infusions compared to 9.8% in placebo-treated groups. Nevertheless, in our clinical experience with Pmab in breast cancer patients, IR frequency was much higher than previously reported. Although the severity of symptoms induced by IR is primarily of grades 1-2, less than 1% of cases are classified as grades 3-4, according to the Common Terminology Criteria for Adverse Events scale ^(6), (7), (8)^.

We are forced to treat patients after IR unexpectedly occurs because no predictive factors for IR on Pmab have yet been established. IR with the first dose of Pmab is not rare; in fact, it is more common in our experience than reported in the literature. Thus, revealing the risk factors for IR and establishing proactive indicators are essential for ensuring patient safety. We investigated IR risk factors in the initial treatment of HER2-positive breast cancer with Pmab-Tmab combination therapy.

## Materials and Methods

### Patients

We retrospectively reviewed the medical records of 59 consecutive breast cancer patients who first received Pmab-Tmab combination therapy with or without chemo- or endocrine therapy at the University of Tokyo Hospital from January 2014 to February 2021. Two patients were excluded because of their regular use of steroids to treat chronic connective tissue disease. Finally, 57 patients were enrolled in our analysis. The protocol of the present study was approved by the Ethics Committee of the University of Tokyo Hospital (approval No. 10144-(3)). Written informed consent was obtained from all patients included in this study.

### Study protocol

Clinical information was reviewed in medical records and nursing records. We explored patient characteristics and clinical data to identify possible risk factors for IR and then analyzed these factors statistically. Clinical data included sex, age, height, body weight, body mass index (BMI), history of allergy, history of smoking, history of alcohol consumption, treatment settings for breast cancer, drugs combined with Pmab and Tmab, history of previous chemotherapy, and baseline blood test data. Those treated within the last three months were also classified by therapeutic drugs used regarding the past chemotherapy treatment history. Blood sampling, which was routinely conducted as a pre-check for a new treatment with Pmab, included white blood cell counts, neutrophil counts, lymphocyte counts, monocyte counts, red blood cell counts (RBC), hemoglobin (Hb), hematocrit (Hct), mean corpuscular volume, mean corpuscular hemoglobin, mean corpuscular hemoglobin concentration, platelet count, neutrophil-to-lymphocyte ratio, monocyte-to-lymphocyte ratio, platelet-to-lymphocyte ratio, aspartate aminotransferase, alanine aminotransferase, alkaline phosphatase (ALP), γ-glutamyl transpeptidase, lactate dehydrogenase, total protein, albumin, blood urea nitrogen, creatinine, and C-reactive protein. The presence or absence of estrogen receptor (ER) expression in breast cancer was also considered. Those with 1% or more ER expression in the tumor as measured by immunohistochemistry were judged to be ER-positive.

The drugs were sequentially administered as Pmab (840 mg) for 60 minutes, Tmab (8 mg/kg) for 90 minutes, followed by supportive care drugs and a chemotherapeutic agent.

The criteria of IR were defined as follows: (a) occurring during or within 24 h after an infusion of Pmab; (b) showing symptoms such as pyrexia (body temperature ≥ 37.6°C), chills, fatigue, hypertension, nausea, vomiting, diarrhea, and cough. We examined the frequency of IR during or within one hour after the initial administration of Pmab-Tmab.

### Statistical analyses

Continuous variables were compared using either Student’s *t-*test or non-parametric Wilcoxon rank-sum tests. Categorical variables were compared using Fisher’s exact test. Bonferroni’s correction was applied for multiple comparisons. Before and after anthracycline-cyclophosphamide (AC) treatment in a patient, differences in Hb levels were analyzed using the Wilcoxon signed-rank test. We conducted a univariate logistic regression analysis to identify a risk factor associated with IR. A receiver-operating characteristic (ROC) curve analysis was performed to determine the best cut-off value for risk factors predicting IR. Statistical significance was set at P < 0.05. All statistical analyses were performed using R (version 4.0.3) ^(9)^.

## Results

### Patient characteristics

Of the 57 patients, 56 were female and one was male. The median age of the patients was 60 years, the median height was 1.56 m, the median body weight was 53.9 kg, and the average BMI was 21.8 kg/m^2^. Twenty-six of the 57 patients (46%) had a history of allergies, 19 (33%) had a history of smoking, and 24 (42%) drank alcohol. The treatment settings were pre-operative therapy in 20 cases (35%), post-operative therapy in 10 (18%), recurrent therapy in 15 (26%), and de novo stage IV treatment in 12 (21%). The drugs combined with Pmab-Tmab were docetaxel for 39 (68%), paclitaxel for five (9%), exemestane for one (2%), anastrozole for one (2%), and none for 11 patients (19%). Forty-five patients (79%) had been treated with cytotoxic chemotherapy before first receiving Pmab-Tmab therapy. Within the last three months before pertuzumab administration, 38 (67%) had received chemotherapy, of which 30 (53%) had been treated with an anthracycline-containing regimen (Table 1).

**Table 1.** Patient Characteristics.

Number of patients		57 (100%)
Gender, n	female	56 (98%)
	male	1 (2%)
Age, years		60.0 (28-84)
Height, m		1.56 (1.45-1.70)
Body weight, kg		53.9 (40.3-100)
BMI, kg/m^2^		21.8 (17.2-40.2)
Histories, n	allergy	26 (46%)
	smoking	19 (33%)
	alcohol consumption	24 (42%)
Treatment settings, n	pre-operative	20 (35%)
	post-operative	10 (18%)
	recurrent	15 (26%)
	stage IV	12 (21%)
Drugs combined with Pmab-Tmab, n	docetaxel	39 (68%)
	paclitaxel	5 (9%)
	capecitabine	1 (2%)
	exemestane	1 (2%)
	none	11 (19%)
Previous history of chemotherapy, n		
any time		45 (79%)
within 3 months	Total	38 (67%)
	anthracycline + cyclophosphamide	30 (53%)
	paclitaxel	1 (2%)
	Tmab	4 (7%)
	Tmab + paclitaxel	1 (2%)
	lapatinib + capecitabine	1 (2%)
	capecitabine	1 (2%)
	
none		12 (21%)
Estrogen receptor, n	positive	36 (63%)
	negative	21 (37%)
IR occurrence, n	yes	25 (44%)
	no	32 (56%)

Data are median value (range) or n (%). *BMI*, body mass index; *Pmab*, Pertuzumab; *Tmab*, Trastuzumab

**Figure 1.**
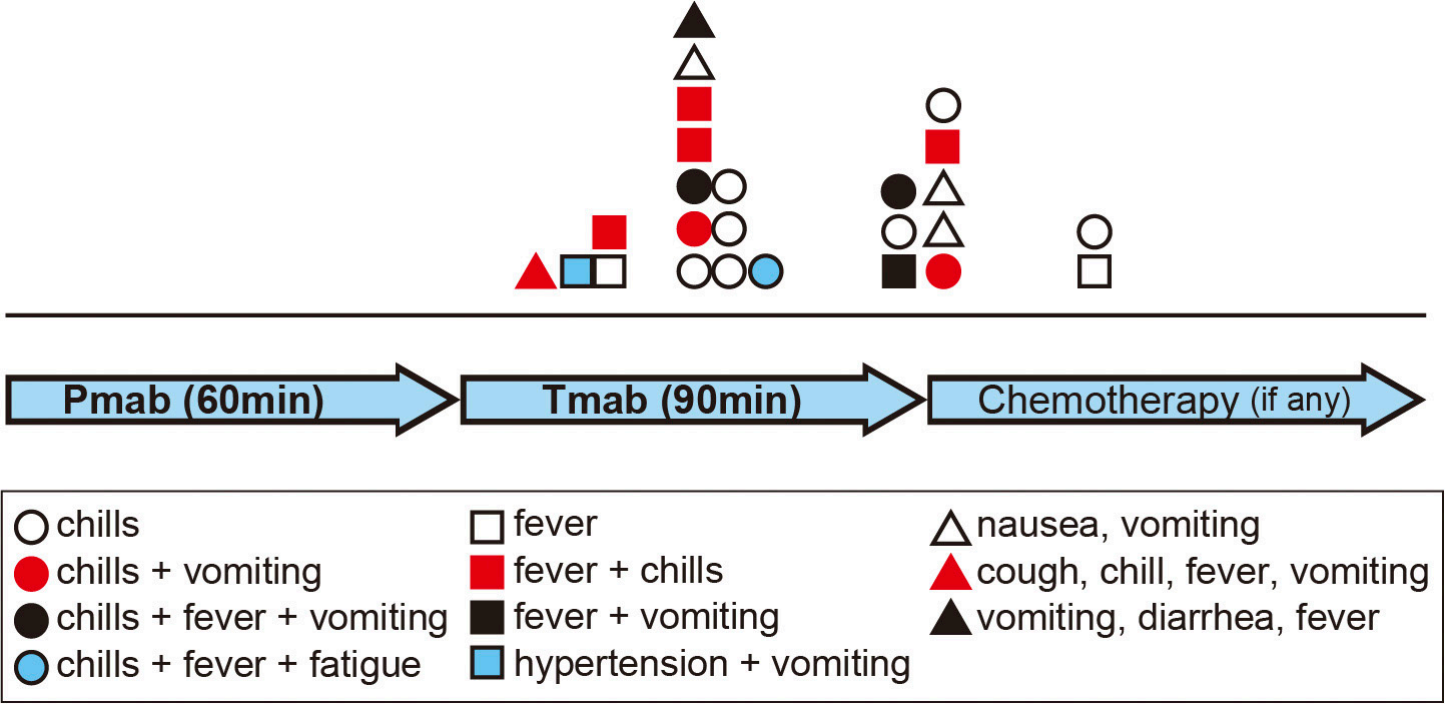
Timing of IR development. IR occurred during Tmab administration in 15 cases; just as administration was being completed or immediately after completion of Tmab administration in eight cases; about one hour after completion of Pmab-Tmab administration in two cases. The symbols indicate the symptoms experienced by patients: chills are indicated by white circles; chills and vomiting by red circles; chills, fever, and vomiting by black circles; chills, fever, and fatigue by a blue circle; fever by white squares; fever and chills by red squares; fever and vomiting by a black square; hypertension and vomiting by a blue square; nausea and vomiting by white triangles; cough, chills, fever, and vomiting by a red triangle; and vomiting, diarrhea, and fever by a black triangle. Pmab, pertuzumab; Tmab, trastuzumab.

The incidence rate of IR was 44% (25/57) (Table 1). In 15 cases, IR occurred during Tmab administration; in eight patients, IR occurred near the end or immediately after completing Tmab administration; and in two cases, it occurred approximately one hour after completion of Pmab-Tmab administration. The time of IR occurrence and the symptoms patients experienced are listed in Figure 1.

### Comparison of the groups with and without IR

When comparing the groups of treatment settings, the frequency of IR occurrence was significantly higher in the post-operative treatment group than in the relapse treatment group (P = 0.031). Univariate analysis revealed that, compared with the patients without IR, those who experienced IR showed significantly lower RBC count (3.47 × 10^6^/μL vs. 4.03 × 10^6^/μL, P < 0.001), lower Hb concentration (10.8 g/dL vs. 12.0 g/dL, P = 0.0011), and lower Hct (32.4% vs. 36.3%, P < 0.001). Body weight (50.0 kg vs. 55.0 kg, P = 0.092) and BMI (20.3 kg/m^2^ vs. 22.4 kg/m^2^, P = 0.058) were relatively lower in the IR-positive group, however the P-values were slightly above 0.05. Also, patients with IR more frequently received AC therapy within the last three months, but the difference was slightly less than significant (P = 0.061) (Table 2).

**Table 2.** Univariate Analysis of Demographic and Clinical Variables between Patients with and without Infusion Reaction (IR).

	Without IR (n = 32)	With IR (n = 25)	P-value
Patient characteristics			
Age, years	52 (28-84)	64 (33-83)	0.16
Height, m	1.56 (1.45-1.68)	1.56 (1.46-1.70)	0.91
Body weight, kg	55 (41-97)	50 (40-100)	0.092
BMI, kg/m^2^	22.4 (17-37)	20.3 (17-40)	0.058
History of allergy, n	16 (50%)	10 (40%)	0.59
History of smoking, n	8 (25%)	11 (44%)	0.30
History of alcohol consumption, n	15 (47%)	9 (36%)	0.43
Estrogen receptor, n			
positive	20 (63%)	16 (64%)	1.0
negative	12 (38%)	9 (36%)	
Treatment settings, n			
pre-operative	11 (34%)	9 (36%)	0.031^*^
post-operative	2 (6%)	8 (32%)	
recurrent	12 (38%)	3 (12%)	
stage IV	7 (22%)	5 (20%)	
Anti-cancer drug administration within 3 months before Pmab, n			
anthracycine	13 (41%)	17 (68%)	0.061
none or other than anthracycline	19 (59%)	8 (32%)	
Blood test data			
WBC, /μL	5200 (2600-15300)	5000 (1700-15700)	0.57
NTR, /μL	3400 (1600-12900)	3500 (850-13500)	0.63
LYM, /μL	870 (370-2290)	860 (200-2340)	0.48
MON, /μL	300 (160-980)	320 (100-1600)	0.76
RBC, 10^4^/μL	400 (300-500)	350 (210-480)	*<0.001*
Hb, g/dL	12.0 (9.6-14)	10.8 (6.5-14)	*0.0011*
Hct, %	36 (30-44)	32 (19-42)	*<0.001*
MCV, fL	93 (81-100)	93 (79-99)	0.61
MCH, pg	31 (24-42)	31 (26-35)	0.49
MCHC, %	33 (30-35)	34 (32-35)	0.21
PLT, 10^4^/μL	27 (9.5-52)	26 (4.6-55)	0.69
NLR	3.9 (1.1-14)	4.0 (1.3-26)	0.86
MLR	0.36 (0.14-1.3)	0.49 (0.11-1.3)	0.37
PLR	300 (110-1110)	300 (140-1000)	0.88
AST, U/L	21 (10-53)	21 (11-420)	0.74
ALT, U/L	17 (5-77)	16 (8-91)	0.64
ALP, U/L	230 (60-650)	240 (95-2900)	0.50
γ-GTP, U/L	28 (18-150)	22 (15-1400)	0.17
LDH, U/L	220 (160-380)	210 (140-1400)	1.0
TP, g/dL	6.9 (6.3-7.5)	6.8 (5.8-7.9)	0.27
Alb, g/dL	4.1 (2.9-4.7)	3.9 (2.4-4.5)	0.11
BUN, mg/dL	12 (7.0-55)	13 (6.1-21)	0.55
Cre, mg/dL	0.59 (0.40-1.80)	0.56 (0.41-0.81)	0.81
CRP, mg/dL	0.090 (0-3.9)	0.070 (0-5.2)	0.52

Data are median value (range) or n (%). ^*^Only the P-value for post-operative vs. recurrent is shown. *BMI*, body mass index; *WBC*, white blood cell; *NTR*, neutrophil; *LYM*, lymphocyte; *MON*, monocyte; *RBC*, red blood cell; *Hb*, hemoglobin; *Hct*, hematocrit; *MCV*, mean corpuscular volume; *MCH*, mean corpuscular hemoglobin; *MCHC*, Mean Corpuscular Hemoglobin Concentration; *Plt*, platelet; *NLR*, neutrophil-to-lymphocyte ratio; *MLR*, monocyte-to-lymphocyte ratio; *PLR*, platelet-to-lymphocyte ratio; *AST*, aspartate aminotransferase; *ALT*, alanine aminotransferase; *ALP*, alkaline phosphatase; *γ-GTP*, γ-glutamyl transpeptidase; *LDH*, lactate dehydrogenase; *TP*, total protein; *Alb*, albumin; *BUN*, blood urea nitrogen; *Cre*, creatinine; *CRP*, C-reactive protein

### Comparison of IR occurrence between groups who did or did not receive AC therapy within the last three months

To investigate which factors could lead to a decrease in erythrocyte levels, we calculated Spearman’s rank-order correlation to analyze the correlation between the indicators of anemia (RBC, Hb, and Hct) and other continuous variables. Of these, when combinations of the anemic factors (such as RBC vs. Hb) were excluded, a coefficient ρ of more than 0.4 or less than −0.4 was obtained only for RBC vs. mean corpuscular volume (ρ = −0.45), RBC vs. mean corpuscular hemoglobin (ρ = −0.43), and Hb vs. neutrophil-to-lymphocyte ratio (ρ = 0.40) (data not shown). From this result, we deduced that no factor among the continuous variables showed a strong correlation with anemic indices.

In contrast, we divided the patients into two groups according to each categorical variable (presence or absence of a history of allergy, smoking, alcohol consumption, and chemotherapy, AC treatment within three months, ER status of the tumor, and whether Pmab was used as a perioperative therapy or for tumor metastasis and relapse). As we compared the indices of anemia between the two groups using the Wilcoxon rank-sum test, only when we classified patients by AC treatment in the last three months, we obtained significant differences in the anemia indicators (RBC: P < 0.001; Hb: P < 0.001; Hct: P < 0.001, data not shown). Hence, anemia was significantly influenced by whether the patient received AC therapy most recently before pertuzumab administration.

### Categorizing the patients according to AC therapy within the last three months

We divided the patient groups according to whether they received AC therapy within the past three months of pertuzumab administration, and examined which factors were associated with the presence or absence of IR in each group. Looking at the group that received AC chemotherapy within the last three months, RBC, Hb, and Hct, being found to be significantly lower in patients experiencing IR than those not experiencing IR, while there was no difference in other factors. In contrast, in the group that was not treated with AC immediately before Pmab treatment, the only factors that showed a significant difference between patients with IR and those without IR were body weight, BMI, and ALP value: there was no difference in RBC, Hb and Hct between the two groups (Table S1a and b).

### Comparison of hemoglobin levels after the first dose of anthracycline and that of pertuzumab in patients who received anthracycline treatment in the previous three months

**Figure 2.**
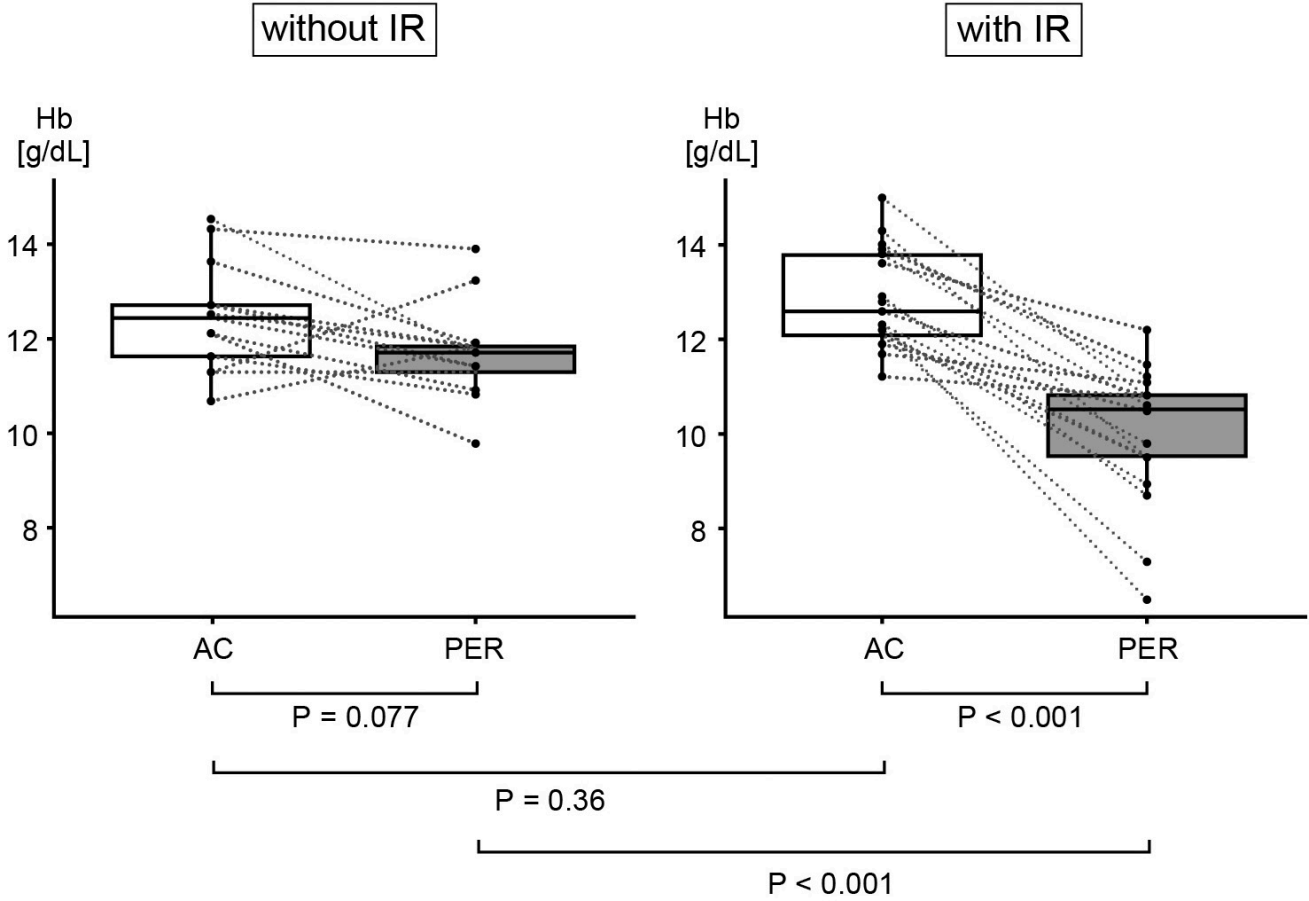
Changes in hemoglobin levels between the first dose of anthracycline and that of pertuzumab in patients who had received anthracycline treatment within the previous three months. At the point of starting AC treatment, no significant difference in Hb level was found between the groups with and without IR (median 12.6 g/dL vs. 12.4 g/dL, P = 0.36). In the group with IR, Hb significantly decreased after completing the AC treatment (median 12.6 g/dL vs. 10.5 g/dL, P < 0.001). Hb tended to decline slightly after AC treatment in the IR-free group, but not significantly (median 12.4 g/dL vs. 11.7 g/dL, P = 0.077). Bars in the boxes represent median values. AC, anthracycline-cyclophosphamide; Hb, hemoglobin; IR, infusion reaction; PER, pertuzumab.

Figure 2 shows the degree of change in hemoglobin levels between the first dose of AC and that of Pmab in the patients who received AC treatment within the last three months. No significant difference in Hb level was identified at the start of AC treatment, between the groups with and without IR (median 12.6 g/dL vs. 12.4 g/dL, P = 0.36). However, in the group with IR, Hb significantly decreased after completing AC treatment (median 12.6 g/dL vs. 10.5 g/dL, P < 0.001). Hb tended to decline slightly after AC treatment in the IR-free group, but this was not a significant difference (median 12.4 g/dL vs. 11.7 g/dL, P = 0.077).

According to univariate logistic regression analysis focusing on Hb, the most significant risk factor for IR was the ratio of Hb value at the first administration of Pmab (Hb (P)) to that at the beginning of AC (Hb (A)) (log odds ratio: −17, 95% confidence interval (CI): 5.0 × 10^−15^-1.1 × 10^−3^), rather than Hb (P) alone (log odds: −1.5, 95% CI: 0.044-0.58, or the difference between Hb (A) and Hb (P) (log odds ratio: −1.3, 95% CI: 0.092-0.61) (Table 3).

**Table 3.** Univariate Logistic Regression Analysis Focusing on Hemoglobin Values.

Variables	Odds ratio	Log odds ratio	95% CI
Hb (P)	0.22	−1.53	[0.044, 0.58]
Hb (P) - Hb (A)	0.29	−1.25	[0.092, 0.61]
Hb (P)/HB (A)	3.5 × 10^−8^	−17.2	[5.0 × 10^−15^, 1.1 × 10^−3^]

*Hb*, hemoglobin *Hb (P)*, hemoglobin value at the first administration of pertuzumab *Hb (A)*, hemoglobin value at the first administration of anthracycline *Hb (P) - Hb (A)*, difference between Hb (P) and Hb (A) *Hb (P)/HB (A)*, ration of Hb (P) to Hb (A) *CI*, confidence interval

**Figure 3.**
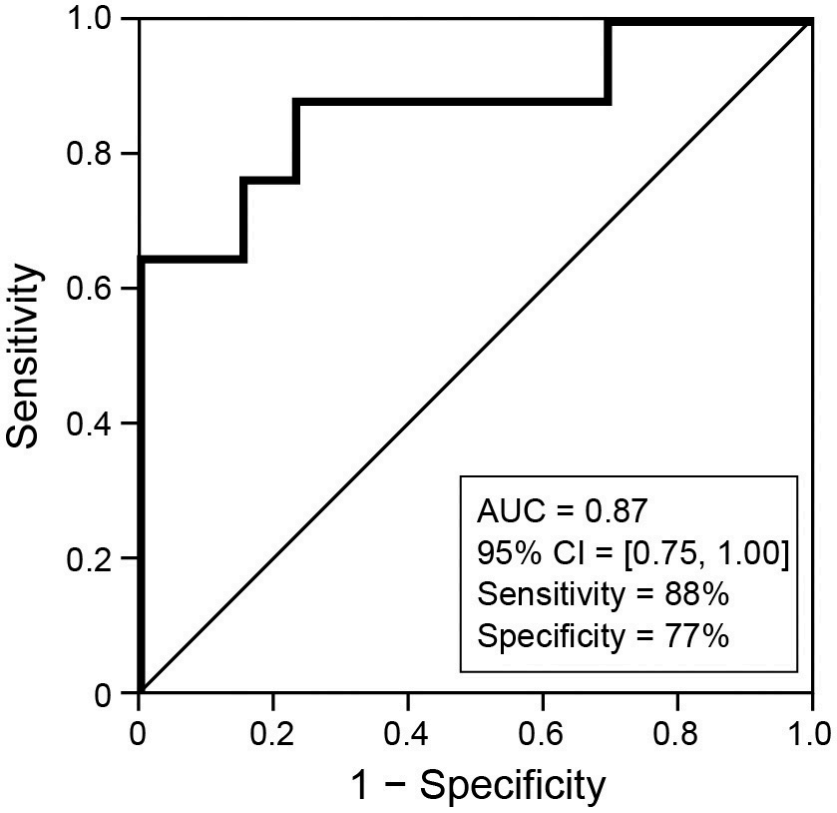
Area under the receiver-operating characteristic curve for IR prediction. When setting the cut-off line of the ratio of Hb (P) to Hb (A) at 0.90, sensitivity was 88% (15/17), and specificity was 77% (10/13) (area under the curve: 0.87; 95% CI: 0.75-1.0). AUC, area under the curve; CI, confidence interval.

Figure 3 shows the result of the ROC analysis. When setting the cut-off line of the ratio of Hb (P) to Hb (A) as 0.90, the sensitivity was 88% (15/17), and the specificity was 77% (10/13) (area under the curve: 0.87; 95% CI: 0.75-1.0).

## Discussion

This study found that IR was more likely to occur after the first dose of pertuzumab in patients with a significant decrease in erythrocyte levels from before to after AC treatment, while further, and specifically, IR happened with a high probability in patients whose Hb level decreased by 10% or more from the start to the end of AC.

IR occurred in 44% of the patients in our study; this rate was much higher than that obtained in the clinical trials of Pmab (13%) ^(6), (8)^. This finding was possibly because the cohort in this study included lots of patients who had been treated with AC immediately before the clinical trials. Many of them had low Hb levels. In our research, most of the patients experienced IR during or at the end of the Tmab infusion. In general, IR symptoms rarely become severe. However, there is consensus that the injection should immediately be discontinued once a reaction is suspected, and appropriate supportive treatment should be given ^(5)^. To safely administer systemic treatment, predicting the risk in advance and preparing to take appropriate action when IR occurs is essential.

To our knowledge, few reports have examined the factors predicting IR. Palomar Coloma and colleagues reported that prior history of allergies, tobacco and alcohol use were associated with cetuximab-related IR ^(10)^. In another report, rituximab infusion-related reactions in adult hematology patients with malignant pathologies were associated with higher body weight, diagnostic differences of hematologic neoplasms, lower hemoglobin, and bone marrow involvement in univariate analysis, while lower hemoglobin barely showed a significant difference in multivariate logistic regression analysis ^(11)^.

Anemia is one of the critical side effects of AC treatment ^(12)^. In this study, erythrocyte-related factors just before the first dose of Pmab were significantly reduced from baseline if there was a history of AC treatment within the past three months, where, in the group of patients who had received AC within the past three months, a simple logistic regression analysis suggested that decreased erythrocyte levels after AC treatment were a risk factor for IR development. We recommend taking prophylactic measures such as anti-inflammatory medication or anti-allergic drugs if a patient’s Hb level is reduced by 10% or more before the first dose of Pmab compared to that before the first dose of AC since Pmab administration is expected to induce IR.

Based on our results, anemia might induce excessive cytokine release to antibody drugs whereby there might be a direct causal relationship between anemia and IR. Alternatively, suppression mechanisms against cytokine release might not work effectively under anemic conditions. Chronic inflammatory states are reported in chronic autoimmune anemia, such as hemolytic anemia ^(13)^. In acute critically ill patients, in addition to direct blood loss due to trauma or surgery, anemia is induced by inflammation. Inflammation, which persists as long as the anemic condition continues ^(14)^, blunts the responsiveness of erythropoietin and causes functional iron deficiency. In contrast, whether anemia itself causes inflammation is poorly understood, saying which, Arthur CM et al. showed that anemia causes increased intestinal tract inflammation and barrier disruption by altering macrophage function in the preterm infant mouse model. According to them, blood levels of IFN-γ increased in preterm infant mice as the severity of anemia increased. Also, phlebotomy-induced hypoxia significantly increased macrophage pro-inflammatory cytokine levels ^(15)^. Although premature infant mice and adult cancer-bearing patients in clinical practice are entirely different, it may be plausible that chronic inflammation and hypoxic conditions of organs associated with chronic anemia facilitate inducing cytokine release after antibody-drug administration. Further study, both of biological and clinical aspects, is warranted.

However, it may be premature to conclude from this study that anemia itself is a direct trigger for the development of IR. Instead, the systemic background of the patients, such as their susceptibility to the side effects of anemia caused by anticancer drug treatment including AC therapy, might be the cause of IR development. Interestingly, in a result that might indicate that chronic anemia does not directly relate to the occurrence of IR, our study revealed no significant difference in erythrocyte-related factors between the patients with and those without IR in the patient cohort that had not undergone previous AC treatment. We infer that an anemic condition itself is not the immediate cause of IR but might play a role as an indicator of the hematopoietic function and an immune system disturbed by AC treatment. Meanwhile, body weight, BMI, and ALP showed significant differences between patients with and without IR in the group without previous AC treatment, where we see that, as the number of cases was small, careful consideration is needed to determine whether these are actual risk factors.

Interestingly, concerning the treatment settings, the post-operative group patients had a significantly higher incidence of IR (8 out of 10) compared to the recurrent treatment group (3 of 15) (P = 0.031). In the post-operative group, where on the one hand only two patients had received AC within three months before Pmab therapy in the recurrent treatment group, on the other hand 8 of 10 patients had received AC within the previous three months, and 7 of them developed IR, a difference suggesting that the presence or absence of AC administration immediately before might be one reason for this difference. However, while 16 of 20 patients received AC within three months in the group that received Pmab as pre-operative therapy, the occurrence of IR was 9/20, relatively lower than the post-operative group. The incidence rates of 10% loss of Hb value due to AC treatment were 13/16 patients in the NAC group and 5/8 patients in the post-operative group, with no significant difference (data not shown). Since the tumor burden in a body is generally considered almost zero immediately after surgery for early-stage breast cancer, these results suggest no relationship between tumor volume and the development of IR. Instead, surgical invasion before Pmab treatment might have altered the patient’s immune system and negatively affected the outcome of IR. In any case, the number of cases is small and additional studies are needed.

Although not shown this time, dose-dense AC and three-week AC regimens did not differ in the frequency of IR occurrence and the degree of anemia, where dose-dense chemotherapy more frequently induces anemia than tri-weekly therapy ^(16), (17)^. Granulocyte-colony stimulating factor, which is generally combined with dose-dense treatment to support myelosuppression, may in some cases exacerbate anemia ^(18)^. In the future, we aim to examine in more cases whether the interval of AC administration and granulocyte-colony stimulating factor support affects the risk of IR induced by pertuzumab.

We do not recommend routine blood transfusions before the start of Pmab in order to prevent IR. Whether it is possible to prevent the onset of IR by correcting the apparent Hb value by blood transfusion needs to be carefully examined in the future. For anemic patients with a high tumor burden, such as recurrent- or stage IV- cases, while the attending physician should carefully determine the indications for transfusion on a case-by-case basis, giving a blood transfusion may be positively considered in parallel with cancer treatment.

This study has some limitations. While to the best of our knowledge, this is the first publication that identified decreased erythrocyte levels after preceding AC treatment as a promising predictive marker for the occurrence of IR in response to Pmab-Tmab treatment, it was nevertheless a retrospective study with a small sample size at a single institute.

The present study showed that the onset of IR after the first dose of Pmab was significantly higher when anemia was clinically present after AC therapy. Still, the mechanism of this phenomenon is unknown, and therefore future investigation into the molecular biological mechanism is warranted. Nevertheless, it is quite easy to determine the risk of developing IR at the first dose of Pmab based on blood sampling in standard clinical practice. From this aspect, we hope that our findings will be validated at other institutions, believing as we do that our findings are significant.

Our study found that the presence of anemia at the start of pertuzumab treatment can predict the risk of developing IR. Especially in the group that had received anthracycline-containing chemotherapy immediately before, there was a strong association between IR occurrence and lowered erythrocyte levels from the patients’ baseline. Resulting in further investigation being warranted to establish evidence that patients at high risk for IR can be identified and adequately managed with preventive treatments, detecting decreased erythrocyte levels after preceding AC treatment harbors a predictive capacity for identifying patients at risk for developing IR in response to Pmab administration.

## Article Information

### Conflicts of Interest

None

### Acknowledgement

We thank Clear Science for the English language editing of this manuscript.

### Author Contributions

KO is the first author and wrote this manuscript. KO, MT designed and managed this study. KO, AM, MH, AS, TN, and KN collected clinical data for this study from patients’ records. MT and YS critically reviewed the manuscript. All authors have read and approved the manuscript.

### Approval by Institutional Review Board (IRB)

The protocol of the present study was approved by the Ethics Committee of the University of Tokyo Hospital (approval No. 10144-(3)). Written informed consent was pre-obtained from all patients included in this study.

### Disclaimer

Yasuyuki Seto is one of the Editors of JMA Journal and on the journal’s Editorial Staff. He was not involved in the editorial evaluation or decision to accept this article for publication at all.

## Supplement

Table S1a

Table S1b
