# Supplementary material for: Exploring Predictive Risk Factors of Infusion Reactions with First Pertuzumab Administration in HER2-positive Breast Cancer Patients: A Single Institution Experience
Source: JMA J. 2022 Dec 23;6(1):63–72. doi: 10.31662/jmaj.2022-0132 (PMC9908404; doi:10.31662/jmaj.2022-0132)
Supplement: Supplementary file 1 — Table S1a [file 2433-3298-6-1-0063-s001.pdf]

Table S1a. Univariate analysis of demographic and clinical variables between patients with and without infusion reaction (IR)(Group receiving anthracycline treatment within the last three months)

	Without IR (n = 13)	With IR (n = 17)	P-value
Patient characteristics			
Age, years	48 (28–75)	61 (33–77)	0.073
Height, m	1.57 (1.48–1.68)	1.56 (1.48–1.70)	0.91
Body weight, kg	50 (41–80)	51 (40–100)	0.78
BMI, kg/m^2	20 (17–29)	20 (17–40)	0.80
History of allergy, n	5 (38%)	6 (35%)	1.0
History of smoking, n	2 (15%)	7 (41%)	0.23
History of alcohol consumption, n	5 (38%)	6 (35%)	1.0
Estrogen receptor, n			
positive	11 (85%)	11 (65%)	0.41
negative	2 (15%)	6 (35%)	
Treatment settings, n			
pre-operative	7 (54%)	9 (53%)	0.40*
post-operative	1 (8%)	7 (41%)	
recurrent	2 (15%)	0 (0%)	
stage IV	3 (23%)	1 (6%)	
Blood test data			
WBC, /μL	4900 (3000–15000)	5300 (2200–16000)	0.73
NTR, /μL	3300 (1900–13000)	3600 (1500–13500)	0.94
LYM, /μL	690 (370–1300)	770 (200–2100)	0.53
MON, /μL	400 (180–980)	400 (170–1100)	0.93
RBC, 10^4/μL	370 (300–420)	330 (210–480)	*< 0.001*
Hb, g/dL	11.7 (9.8–13.9)	10.5 (6.5–12.2)	*< 0.001*
Hct, %	35 (30–40)	32 (19–36)	*0.0013*
MCV, fL	94 (84–99)	93 (87–99)	0.66
MCH, pg	32 (28–42)	31 (29–34)	0.83
MCHC, %	33 (32–35)	34 (32–35)	0.89
PLT, 10^4/μL	28 (9.5–50)	31 (4.6–55)	0.70
NLR	5.3 (2.5–14)	5.7 (1.5–26)	0.75
MLR	0.67 (0.23–1.3)	0.56 (0.17–1.3)	0.70
PLR	350 (120–1100)	440 (190–1000)	0.77
AST, U/L	21 (15–53)	20 (11–50)	0.41
ALT, U/L	18 (12–55)	15 (8–66)	0.10
ALP, U/L	300 (110–390)	240 (95–420)	0.14
γ-GTP, U/L	50 (21–150)	22 (15–170)	0.054
LDH, U/L	210 (160–350)	210 (140–310)	0.95
TP, g/dL	6.8 (6.3–7.5)	6.7 (5.8–7.9)	0.30
Alb, g/dL	4.1 (3.7–4.5)	3.9 (3.1–4.5)	0.18
BUN, mg/dL	11 (8.1–19)	13 (6.1–18)	0.32
Cre, mg/dL	0.55 (0.45–0.97)	0.55 (0.41–0.81)	0.78
CRP, mg/dL	0.040 (0–0.37)	0.065 (0–2.3)	0.088

Data are median value (range) or n (%).

*Only the p-value for post-operative vs. recurrent is shown.

BMI , body mass index; *WBC* , white blood cell; *NTR* , neutrophil; *LYM* , lymphocyte; *MON* , monocyte; *RBC* , red blood cell; *Hb* , hemoglobin; *Hct* , hematocrit; *MCV* , mean corpuscular volume; *MCH* , mean corpuscular hemoglobin; *MCHC* , Mean Corpuscular Hemoglobin Consentration; *Plt* , platelet; *NLR* , neutrophil-to-lymphocyte ratio; *MLR* , monocyte-to-lymphocyte ratio; *PLR* , platelet-to-lymphocyte ratio; *AST* , aspartate aminotransferase; *ALT* , alanine aminotransferase; *ALP* , alkaline phosphatase; *γ-GTP* , γ-glutamyl transpeptidase; *LDH* , lactate dehydrogenase; *TP* , total protein; *Alb* , albumin; *BUN* , blood urea nitrogen; *Cre* , creatinine; *CRP* , C-reactive protein
