# Supplementary material for: Exploring Predictive Risk Factors of Infusion Reactions with First Pertuzumab Administration in HER2-positive Breast Cancer Patients: A Single Institution Experience
Source: JMA J. 2022 Dec 23;6(1):63–72. doi: 10.31662/jmaj.2022-0132 (PMC9908404; doi:10.31662/jmaj.2022-0132)
Supplement: Supplementary file 2 — Table S1b [file 2433-3298-6-1-0063-s002.pdf]

Table S1b. Univariate analysis of demographic and clinical variables between patients with and without infusion reaction (IR) (Group that had not received anthracycline treatment within the last three months)

	Without IR (n = 19)	With IR (n = 8)	P-value
Patient characteristics			
Age, years	56 (37–84)	66 (58–83)	0.29
Height, m	1.55 (1.45–1.65)	1.56 (1.46–1.63)	0.93
Body weight, kg	57 (41–97)	49 (43–59)	*0.016*
BMI, kg/m^2	24 (18–37)	20 (9–24)	*0.019*
History of allergy, n	11 (58%)	4 (50%)	1.0
History of smoking, n	6 (32%)	4 (50%)	0.44
History of alcohol consumption, n	10 (53%)	3 (38%)	0.68
Estrogen receptor, n			
positive	9 (47%)	5 (63%)	0.68
negative	10 (53%)	3 (38%)	
Treatment settings, n			
pre-operative	4 (21%)	0 (0%)	1.0
post-operative	1 (5%)	1 (13%)	
recurrent	10 (53%)	3 (38%)	
stage IV	4 (21%)	4 (50%)	
Blood test data			
WBC, /µL	5300 (2600–8000)	4100 (1700–11000)	0.25
NTR, /µL	3500 (1600–6300)	2900 (850–8100)	0.27
LYM, /µL	1200 (440–2300)	1100 (670–2300)	0.73
MON, /µL	300 (160–500)	260 (100–1600)	0.27
RBC, 10^4/µL	420 (330–500)	410 (350–480)	0.61
Hb, g/dL	13.1 (9.6–13.8)	13.0 (8.9–14.1)	0.99
Hct, %	39 (31–44)	38 (28–42)	0.52
MCV, fL	92 (81–100)	91 (79–99)	0.44
MCH, pg	31 (24–34)	31 (26–35)	0.85
MCHC, %	33 (30–35)	34 (32–35)	0.12
PLT, 10^4/µL	27 (20–52)	21 (15–39)	0.058
NLR	2.9 (1.1–9.0)	2.4 (1.3–4.8)	0.55
MLR	0.23 (0.14–0.73)	0.22 (0.11–0.94)	0.89
PLR	230 (110–650)	180 (140–310)	0.47
AST, U/L	21 (10–45)	22 (16–420)	0.65
ALT, U/L	16 (5–77)	18 (11–91)	0.43
ALP, U/L	86 (60–650)	250 (110–2900)	*0.029*
γ-GTP, U/L	24 (18–33)	21 (15–1400)	0.78
LDH, U/L	220 (180–380)	240 (150–1400)	0.86
TP, g/dL	7.0 (6.4–7.4)	7.0 (6.0–7.7)	0.18
Alb, g/dL	4.1 (2.9–4.7)	4.0 (2.4–4.1)	0.18
BUN, mg/dL	13 (7.0–55)	12 (11–21)	0.65
Cre, mg/dL	0.60 (0.40–1.8)	0.61 (0.45–0.77)	0.76
CRP, mg/dL	0.27 (0–3.9)	0.32 (0.020–5.2)	0.80

Data are median value (range) or n (%).
BMI , body mass index; *WBC* , white blood cell; *NTR* , neutrophil; *LYM* , lymphocyte; *MON* , monocyte; *RBC* , red blood cell; *Hb* , hemoglobin; *Hct* , hematocrit; *MCV* , mean corpuscular volume; *MCH* , mean corpuscular hemoglobin; *MCHC* , Mean Corpuscular Hemoglobin Consentration; *Plt* , platelet; *NLR* , neutrophil-to-lymphocyte ratio; *MLR* , monocyte-to-lymphocyte ratio; *PLR* , platelet-to-lymphocyte ratio; *AST* , aspartate aminotransferase; *ALT* , alanine aminotransferase; *ALP* , alkaline phosphatase; *γ-GTP* , γ-glutamyl transpeptidase; *LDH* , lactate dehydrogenase; *TP* , total protein; *Alb* , albumin; *BUN* , blood urea nitrogen; *Cre* , creatinine; *CRP* , C-reactive protein
